# A patient with myasthenia gravis showing lower sensitivity to rocuronium and earlier recovery of train-of-four responses on electromyography compared to acceleromyography: a case report

**DOI:** 10.1186/s40981-025-00803-x

**Published:** 2025-07-07

**Authors:** Yoshiko Murakami, Masafumi Fujimoto, Naoyuki Hirata

**Affiliations:** 1 Department of Anesthesiology, Kumamoto University Hospital, 1-1-1, Honjyo, Cyuoku, Kumamoto‑City, 860-8556 Japan; 2 Department of Anesthesiology, Kumamoto University School of Medical Sciences, Kumamoto University Hospital, 1-1-1, Honjyo, Cyuoku, Honjyo, Kumamoto, 860-8556 Japan

**Keywords:** Myasthenia gravis, Electromyography, Acceleromyography

## Abstract

**Background:**

Although newly developed electromyographic devices have been introduced in anesthetic practice, reports on their use in patients with myasthenia gravis (MG) are lacking. We describe electromyographic monitoring combined with acceleromyography in a myasthenic patient.

**Case presentation:**

A 55-year-old female underwent robot-assisted thoracoscopic thymothymectomy due to MG associated with thymoma. At general anesthesia induction, 0.13 mg/kg of rocuronium completely suppressed the acceleromyographic train-of-four (TOF) response, enabling tracheal intubation. However, the electromyographic TOF count remained at 4. Intraoperatively, rocuronium was administered whenever the acceleromyographic TOF count reached 1, which was consistently delayed compared to the electromyographic TOF count of 1. After surgery, sugammadex 2 mg/kg was administered following confirmation of a TOF count of 2 on both monitors, which enabled successful extubation in the operating room.

**Conclusions:**

This case suggests that combining electromyography with acceleromyography might be more beneficial than electromyography or acceleromyography alone in myasthenic patients, until further evidence is available.

## Background

Recently, newly developed electromyographic devices have been introduced in anesthetic practice, with increasing accessibility through their miniaturization and integration of the specialized sensors designed for ulnar nerve stimulation and quantitative neuromuscular monitoring. However, use of these new electromyographic monitoring devices also has a major limitation: the lack of reports on their use in patients with neuromuscular disorders.

Here, we report a case of myasthenia gravis (MG) in which a newly developed electromyographic device was applied in combination with acceleromyography, a commonly used monitoring method, for routine clinical practice rather than for research purposes.

## Case presentation

A 55-year-old female (159 cm, 76 kg) was scheduled for robot-assisted thoracoscopic thymothymectomy due to MG associated with thymoma. She had developed ptosis and muscle weakness 5 months earlier, and was diagnosed with MG based on the presence of anti-acetylcholine receptor antibodies, a positive edrophonium test, and a decremental response to repetitive nerve stimulation of the ulnar and facial nerves. Prior to surgery, she received high-dose intravenous immunoglobulin therapy, followed by initiation of oral pyridostigmine 180 mg/day, prednisolone 10 mg/day, and tacrolimus 3 mg/day. Her medical history also included hypertension and hyperlipidemia. Preoperative spirometry tests indicated normal pulmonary function with percent vital capacity: 93.5%, forced expiratory volume in 1 s: 2.26 L, and blood test results were within the normal range.

Premedication was avoided before the surgery. Upon her entry into the operating room, routine monitoring was applied, including electrocardiography, pulse oximetry, non-invasive blood pressure (on the right arm), and anesthetic depth monitoring (BISx module NK^®^, Nihon Kohden, Tokyo, Japan). After an intravenous cannula was inserted into the left forearm vein for the administration of drugs, the patient was placed in the supine position with both arms abducted, and a continuous infusion of remifentanil was commenced. General anesthesia was induced with propofol, followed by insertion of a supraglottic airway without neuromuscular blockade and maintained with continuous infusion of propofol for maintaining Bispectral index between 40 and 60.

An acceleromyograph (AF-101P^®^, Nihon Kohden) was placed on the left arm for neuromuscular monitoring at the adductor pollicis (AP) muscle. The acceleromyographic neuromuscular data were transferred in real-time to a patient monitor and recorded via an Anesthesia Information Management System (AIMS; Surgical Support System CAP-2500^®^, Nihon Kohden). Neuromuscular monitoring was performed in the manner recommended in Good Clinical Research Practice guidelines [1]. Train-of-four (TOF) stimulation of the ulnar nerve was applied every 15 s until a stable TOF response was achieved. During this period, another intravenous cannula was inserted into the right forearm vein, and a catheter was placed into the right radial artery for arterial pressure monitoring. Thereafter, an electromyograph and display unit (AF-201P^®^ and VA-201R^®^, Nihon Kohden) were also placed on the right arm. A single-use surface electrode for the AF-201P (NM-345Y^®^, Nihon Kohden) was placed to stimulate the ulnar nerve, with the sensing electrode placed on the abductor digiti minimi (ADM) muscle, according to the manufacturer’s instructions. The electromyographic neuromuscular data were also transferred online to a computer and recorded in real-time using TOF-Watch^®^ SX monitor (MSD Inc., Tokyo, Japan).

**Fig. 1:**
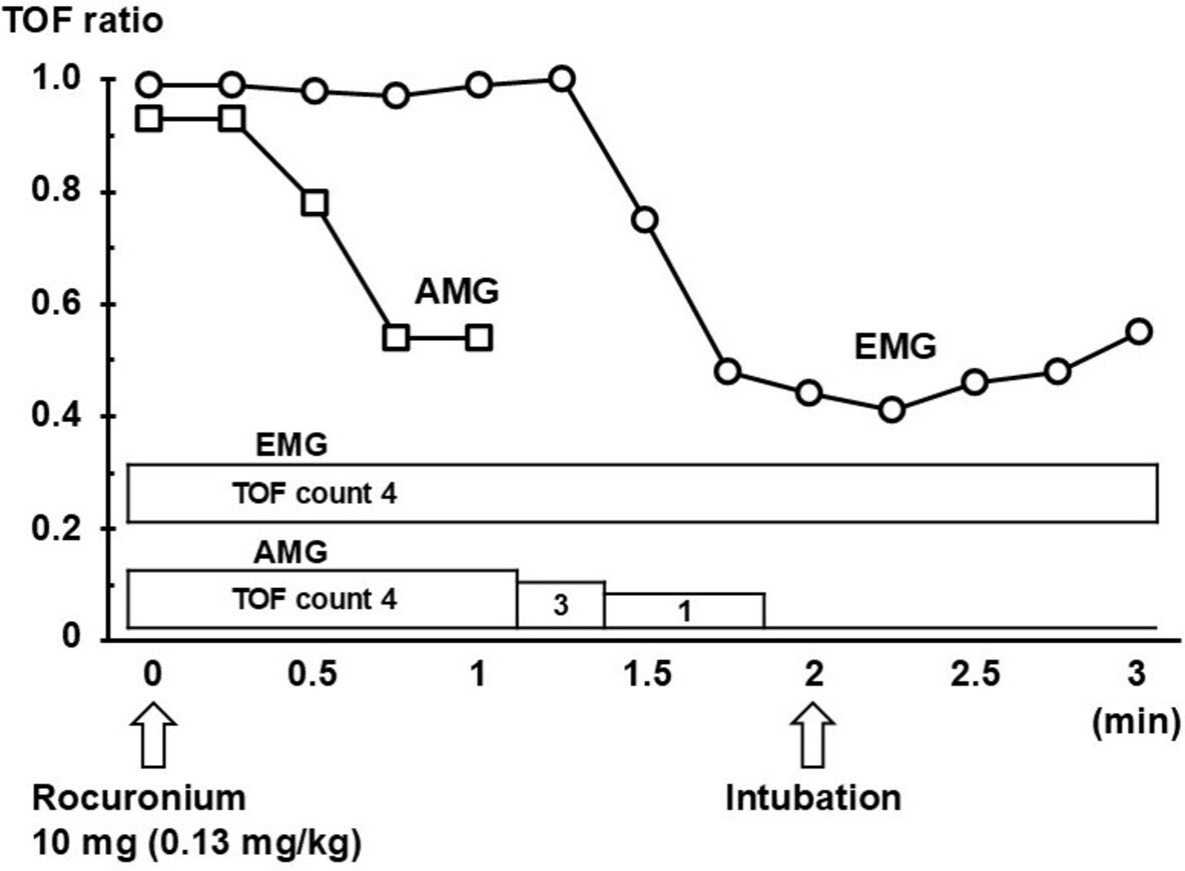
Train-of-four response at the initial administration of rocuronium. Note that electromyographic (EMG) train-of-four (TOF) ratio remained at 1.0 after decrease of acceleromyographic (AMG) TOF ratio 1 min after administration of rocuronium 10 mg. TOF count by EMG was 4 after complete suppression of TOF by AMG. The TOF ratios shown in this figure are not normalized. TOF: train-of-four, AMG: acceleromyography, EMG: electromyography

Subsequently, the built-in calibration function ensured calibration and supramaximal stimulation of the acceleromyograph on the left hand. Due to the specifications of the AIMS, however, setting data such as the stimulating current and the sensitivity of the acceleration transducer after calibration were not recorded. Supramaximal stimulation of the electromyograph on the right hand was also automatically ensured by the built-in calibration function, resulting in a supramaximal current of 39 mA. The control value of the compound muscle action potential was 12.1 mV, and the detection threshold was manually configured to 5% of this value. After confirming stable baseline TOF responses on both monitors, synchronized TOF stimulation was performed with both devices at 15-s intervals, followed by the administration of 10 mg of rocuronium. Baseline TOF ratios just before the rocuronium administration were 0.93 and 0.98 on acceleromyography and electromyography, respectively. The acceleromyographic TOF response was completely blocked immediately after the initial rocuronium administration. Tracheal intubation was then performed without difficulty, and the intubating conditions were rated excellent according to Cooper’s criteria [1, 2]. In contrast, the electromyographic TOF count remained at 4 at this time (Fig. 1).

**Fig. 2:**
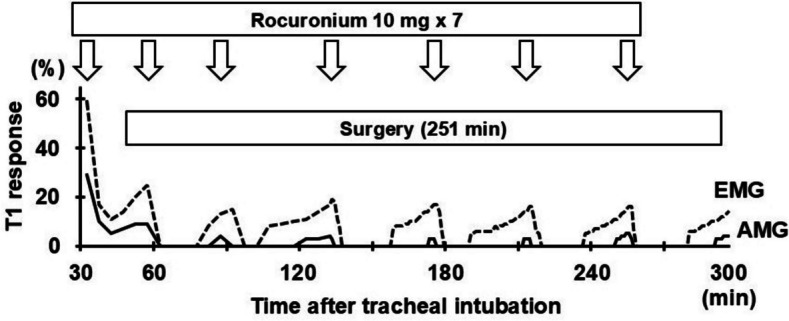
Electromyographic and acceleromyographic first twitch response to train-of-four stimulation during surgery. Note the more rapid recovery and higher amplitude of electromyographic (EMG) T1 response than acceleromyography (AMG) after rocuronium administration. T1 responses shown in this figure are expressed as a percentage of the T1 response before initial rocuronium administration. The solid and broken lines indicate the acceleromyographic and electromyographic T1 responses, respectively. TOF: train-of-four, T1: first twitch response to TOF stimulation, AMG: acceleromyography, EMG: electromyography

At the start of surgery, two additional doses of 10 mg rocuronium were administered, and a TOF count of 0 was confirmed on both monitors. During surgery, synchronized TOF stimuli were delivered by both monitoring devices at 5-min intervals (every 1 min while the robot was docked with the patient). Additional rocuronium doses were administered repeatedly each time the first twitch response to TOF stimulation (T1) was observed on acceleromyography. In the present case, this consistently occurred approximately 20 min later than reappearance of the electromyographic T1 response (Fig. 2). All operative procedures were completed without complications in 251 min, during which further neuromuscular blockade was not requested by the surgeons, and a total of 80 mg of rocuronium was administered.

**Fig. 3:**
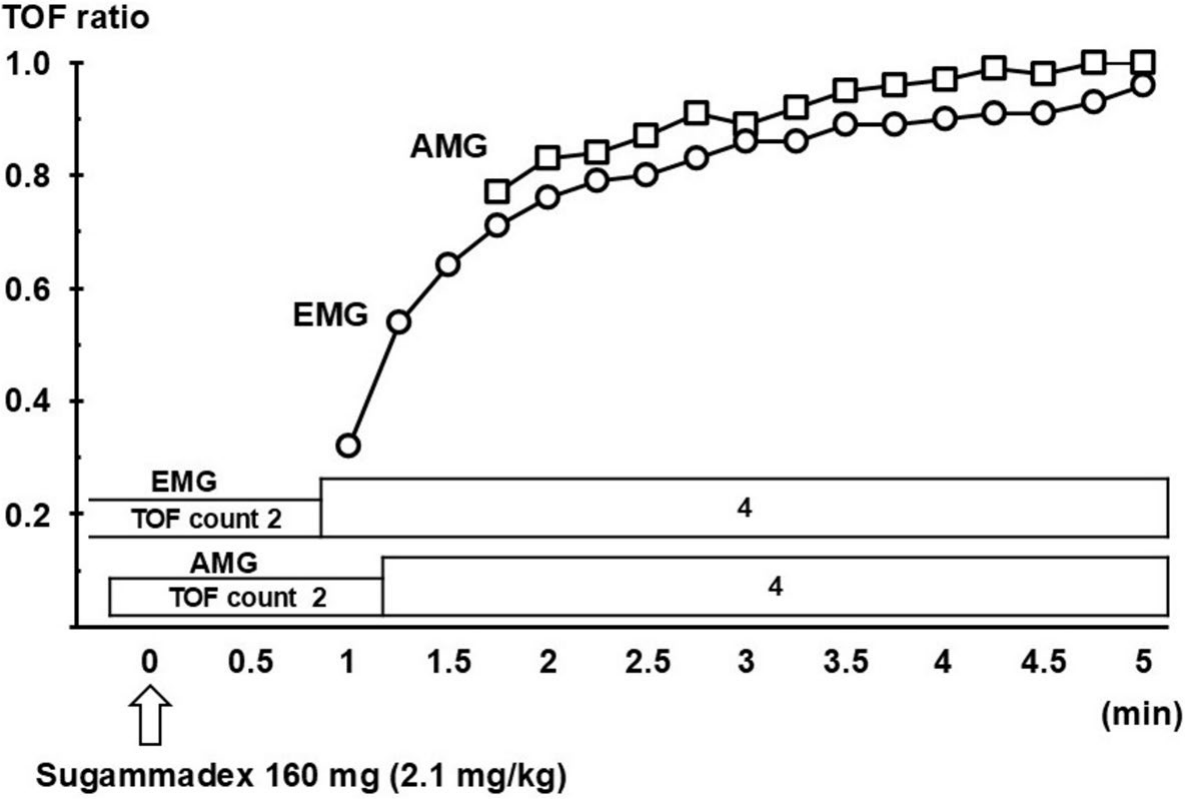
Recovery of train-of-four response after administration of sugammadex. Recovery of train-of-four (TOF) ratio by acceleromyography (AMG) was slower than by electromyography (EMG). The TOF ratios shown in this figure are not normalized. TOF: train-of-four, AMG: acceleromyography, EMG: electromyography

At the end of surgery, the electromyographic TOF count had already reached 2. However, we administered 160 mg of sugammadex only after a TOF count of 2 was also confirmed with acceleromyography. Thereafter, the TOF ratios measured by both monitors rapidly recovered to 90% of their respective baseline values (Fig. 3). The administration of anesthetic agents was then discontinued, and the patient was extubated in the operating room after observation of sufficient recovery of consciousness and spontaneous respiration. Although continuous peripheral surface temperature monitoring was not performed during anesthesia, rectal temperature was continuously measured and kept between 35.7 °C and 36.2 °C using a forced-air warming device. The patient’s postoperative course was uneventful, and no respiratory or other complications suggestive of residual neuromuscular blockade or recurarization were observed.

## Discussion

The use of neuromuscular blocking agents should be avoided in MG patients whenever possible, as they exhibit marked sensitivity to these agents, often resulting in unpredictable or prolonged neuromuscular blockade [3]. However, neuromuscular blocking agents are known to improve surgical conditions not only during laparoscopic surgery, but also during thoracoscopic surgery [4]. Moreover, robotic surgery, in particular, requires secure immobilization of the patient to ensure safety, as accessing the patient becomes difficult once the robot is docked with the patient, and movement by the patient can lead to iatrogenic injury. In the present case, therefore, we planned to use rocuronium with utmost caution under strict neuromuscular monitoring, and to reverse the rocuronium-induced neuromuscular blockade with sugammadex at the end of surgery.

Although the current guidelines for neuromuscular monitoring do not specifically address the preferred neuromuscular monitoring method, whether acceleromyography or electromyography [5, 6], it has been demonstrated that electromyographic neuromuscular monitoring is less variable than acceleromyography and is similar to mechanomyography [7], which is considered the “gold standard” method of neuromuscular monitoring [8]. Additionally, the ability to obtain reliable measurements even when the arms and hands are tucked and access to patients is limited due to surgical positioning is one of the advantages of electromyography. Therefore, electromyography is considered suitable for neuromuscular monitoring during robotic surgery. However, to the best of our knowledge, no studies comparing acceleromyography and electromyography in patients with MG have been reported. In this case, the patient’s position during surgery did not interfere with the setup of acceleromyography. Although electromyography was also used, acceleromyography was chosen for neuromuscular management due to the wealth of our own clinical experience with this device [9].

When using acceleromyography, normalization (dividing the measured TOF ratio by the baseline value) is generally recommended, as the baseline TOF ratio obtained with acceleromyography is often greater than 1.0 [5, 10]. This requires neuromuscular monitoring at the beginning of general anesthesia induction, although it can complicate the process of induction. On the other hand, electromyography does not require normalization, which is another of its advantages. However, even when using electromyography, neuromuscular monitoring at the induction of general anesthesia is still essential in patients with MG, because the dose of neuromuscular blocking agents should be carefully titrated under neuromuscular monitoring.

A decreased baseline TOF ratio, defined as less than 1.0 with acceleromyography at AP muscle and less than 0.9 with electromyography at hypothenar muscles (i.e., the ADM, flexor digiti minimi brevis, and opponens digiti minimi), has been reported as a determinant of an increased response to neuromuscular blocking agents [9, 11]. In the present case, the acceleromyographic baseline TOF ratio was decreased and an increased response to rocuronium was observed at the induction of general anesthesia, as seen by the fact that a much smaller-than-usual dose resulted in complete neuromuscular blockade. In contrast, neither a decreased baseline TOF ratio nor an increased response was observed with electromyographic monitoring at the ADM muscle. Our case report suggests that recognition of the potential for such a discordance between monitoring methods is important.

An investigation of ipsilateral and simultaneous comparisons of TOF responses at the AP muscle using acceleromyography and electromyography in a general surgical population has shown that acceleromyography is more sensitive in detecting early neuromuscular recovery [12]. It has also been reported that the TOF response measured by acceleromyography at the AP muscle is greater and recovers more rapidly than that measured by electromyography at the first dorsal interosseus muscle [13]. Similarly, when acceleromyographic TOF responses at the AP muscle are compared with electromyographic TOF responses at the ADM muscle, the former are generally reported to recover more rapidly [14]. Contrary to these previous reports, however, the earlier recovery of the electromyographic TOF responses was observed in the present case. Had neuromuscular management been based solely on electromyography, the amount of rocuronium administered in the present case would likely have increased, potentially resulting in increased morbidity and the need for postoperative mechanical ventilation [15].

Sugammadex, administered at appropriate doses based on neuromuscular monitoring, similar to those in patients without MG, has been reported to be effective for reversal of rocuronium-induced neuromuscular blockade even in patients with MG [16, 17]. However, the recommended dose of sugammadex (2 mg/kg at a TOF count of 2) is based on studies using acceleromyography [18–20], and the appropriate dose of sugammadex based on electromyography is uncertain. Furthermore, in the present case, the acceleromyographic TOF count recovered to 2 later than recovery of the electromyographic TOF count to 2. Therefore, 2 mg/kg of sugammadex would have been insufficient if its administration was guided only by an electromyographic TOF count of 2. Thus, we recommend using electromyography in combination with acceleromyography when using sugammadex in MG patients, at least until further evidence clarifying the difference in neuromuscular recovery of myasthenic patients as measured by acceleromyography and electromyography becomes available.

While the AP muscle is usually used for acceleromyographic neuromuscular monitoring, not only the AP but also the ADM and other muscles are used for electromyographic neuromuscular monitoring. Since different muscles may be affected in each patient with MG [21], the results of neuromuscular assessment can vary depending on the monitoring methods used. This may be one of the possible explanations for the discordance in responses to neuromuscular blockade between monitoring methods and the contradictory finding regarding neuromuscular recovery in the present case, which contrasts with previous reports.

Additionally, in MG, the difference in the electrical current used for supramaximal stimulation between acceleromyography and electromyography may also influence the results of neuromuscular assessment. Acceleromyography has been reported to require a higher electrical current than electromyography to obtain adequate twitch responses for the acceleration transducer in certain populations with limited thumb movement, such as pediatric patients [22]. As with these patients, in myasthenic patients, the supramaximal current required for acceleromyography is expected to be higher than that for electromyography. When the electrical current increases, muscle activation may increase, causing muscle fatigue and weakening neuromuscular transmission. Furthermore, prior repetitive stimulations to stabilize acceleromyographic TOF responses might also have caused latent muscle fatigue in the present case. However, the critical data related to acceleromyography, such as the stimulating current and the sensitivity of the acceleration transducer after calibration, are unavailable in the present case. This is one of the limitations of this case report. The lack of peripheral surface temperature measurements is also another limitation of this case report. It is known that electromyographic amplitude increases as surface temperature decreases [23, 24]. Further well-designed prospective investigations are required.

In conclusion, this is the first case report comparing electromyography with acceleromyography for neuromuscular monitoring in a patient with MG, highlighting important considerations when using electromyography in such patients. This case might contribute to accumulating clinical evidence on new electromyographic devices in patients with MG, for which further data are needed.

## Data Availability

The datasets related to neuromuscular monitoring in this case report are available from the corresponding author upon reasonable request.

## Abbreviations

MG: Myasthenia gravis
AP: Adductor pollicis
AIMS: Anesthesia Information Management System
ADM: Abductor digiti minimi
TOF: Train-of-four
T1: First twitch response to TOF stimulation
AMG: Acceleromyography
EMG: Electromyography
